# Supplementary material for: Cyanobacterial antimetabolite 7-deoxy-sedoheptulose blocks the shikimate pathway to inhibit the growth of prototrophic organisms
Source: Nat Commun. 2019 Feb 1;10:545. doi: 10.1038/s41467-019-08476-8 (PMC6358636; doi:10.1038/s41467-019-08476-8)
Supplement: Supplementary file 1 — Supplementary Information [file 41467_2019_8476_MOESM1_ESM.pdf]

Supplementary Information for:

Cyanobacterial antimetabolite 7-deoxy-sedoheptulose blocks the shikimate pathway to inhibit the growth of prototrophic organisms

Brilisauer et al.

Supplementary Table 1: Physicochemical data of 7dSh (1) and 5-deoxy-D-ribose (2).

Name	**7-Deoxy-D-*altro*-heptulose (1)**<br>**7-Deoxy-D-sedoheptulose**	**5-Deoxy-D-ribose (2)**
Appearance	white solid	white oil
Sum formula	$C_7H_{14}O_6$ (M_R = 194.18)	$C_5H_{10}O_4$ (M_R = 134.13)
TLC (Rf, colour reaction)	R_f = 0.58 ($CHCl_3$:MeOH 8:5), Orcinol	R_f = 0.71 ($CHCl_3$:MeOH 8:5), Orcinol
HR ESI(+) MS	Calculated for $C_7H_{14}O_6$: 194.0785, found: 217.0688 $[M+Na]^+$ Δ 1.0 ppm	Calculated for $C_5H_{10}O_4$: 134.0574, found: 157.0474 $[M+Na]^+$ Δ 1.5 ppm
Optical rotation value	$[\alpha]_D^{25}$= +11° (*c* = 0.1 in MeOH)	$[\alpha]_D^{25}$= +35° (*c* = 0.1 in MeOH)
^{1}H-NMR	^{1}H-NMR (D_2O, 298 K, 600 MHz):<br>δ = 1.23 (d, 7-H, *J* = 6.6 Hz, 3H), 3.58 (d, 1-H, *J* = 12.2 Hz, 2H), 3.72 (dd, 5-H, *J* = 7.5, 4.4 Hz, 1H), 3.97 (dd, 6-H, *J* = 6.6, 4.4 Hz, 1H), 4.10 (d, 3-H, *J* = 7.8 Hz, 1H), 4.24 (dd, 4-H, *J* = 7.8, 7.5 Hz, 1H) ppm.<br>^{1}H-NMR (MeOD, 25 °C, 600 MHz):<br>δ = 1.22 (s, 7-H, *J* = 6.6 Hz, 3H), 3.50 (s, 1-H, *J* = 11.48 Hz, 2H), 3.65 (dd, 5-H, *J* = 6.5, 4.1 Hz, 1H), 3.90 (dd, 6-H, *J* = 6.6, 4.1 Hz, 1H), 4.02 (d,3-H, *J* = 7.4 Hz, 1H), 4.23 (dd, 4-H, *J* = 7.4, 6.5 Hz, 1H) ppm.	^{1}H-NMR (D_2O, 298 K, 400 MHz):<br>δ = 1.35/1.26 (d, 5-H, *J* = 6.1/6.5 Hz, 3H), 4.01/4.16 (m, 2-H, 1H), 4.01/3.83 (m/t, 3-H, *J* = 5.7 Hz, 1H), 4.01/4.16 (m, 4-H, 1H), 5.21/5.38 (d, 1-H, *J* = 1.7/4.3 Hz, 1H) ppm.<br>^{1}H-NMR (MeOD, 25 °C, 600 MHz):<br>δ = 1.30/1.21 (d, 5-H, *J* = 6.3 Hz, 3H), 3.81/3.98 (dd, 2-H, *J* = 1.0, 4.7/4.3, 5.7 Hz, 1H), 3.58/3.82 (dd, 3-H, *J* = 5.7, 5.7/4.7, 6.3 Hz, 1H), 3.89/4.00 (dd, 4-H, *J* = 4.3, 5.7/6.3. 6.3 Hz, 1H), 5.06/5.22 (d, 1-H, *J* =1.0/4.3 Hz, 1H) ppm.
^{13}C-NMR	^{13}C-NMR (D_2O, 298 K, 150.9 MHz):<br>δ = 17.0 (C-7), 62.5 (C-1), 67.6 (C-6), 74.6 (C-4), 75.8 (C-3), 83.5 (C-5), 101.3 (C-2) ppm.	^{13}C-NMR (D_2O, 298 K, 100.6 MHz):<br>δ = 19.1/17.9 (C-5), 75.2/74.9 (C-3), 75.5/70.4 (C-2), 78.2/78.1 (C-4), 100.8/95.7 (C-1) ppm.

Data are obtained from synthesis products.

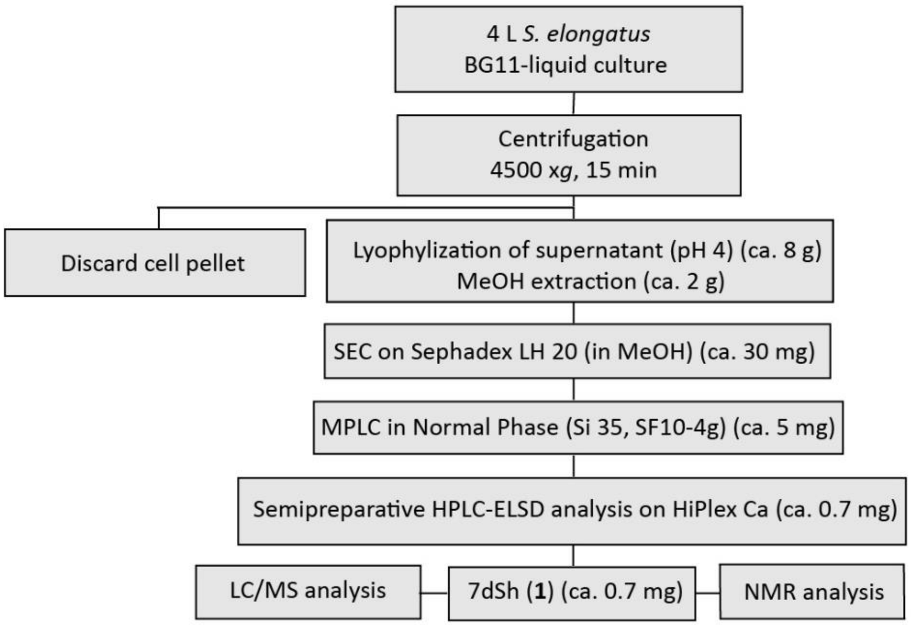

Supplementary Figure 1*:* Isolation scheme for bioactivity-guided purification of 7dSh from *S. elongatus*
The high polarity of 7dSh (**1**) required an optimization of the purification steps from the culture extract prior to final HPLC isolation. Purity was monitored via HPLC-UV-ELSD, NMR and LC-HRMS. HPLC on the ligand/ion-exchange HPLC column HiPlex Ca (Agilent) led to chromatographically pure 7dSh. The yield of the bioactive fractions in each step is indicated in parentheses. Abbreviations: MeOH, methanol; SEC, size-exclusion chromatography; MPLC, medium-pressure liquid chromatography; HPLC, high-performance liquid chromatography; ELSD, evaporative light-scattering detection; LC/MS, liquid chromatography mass spectrometry; NMR, nuclear magnetic resonance.

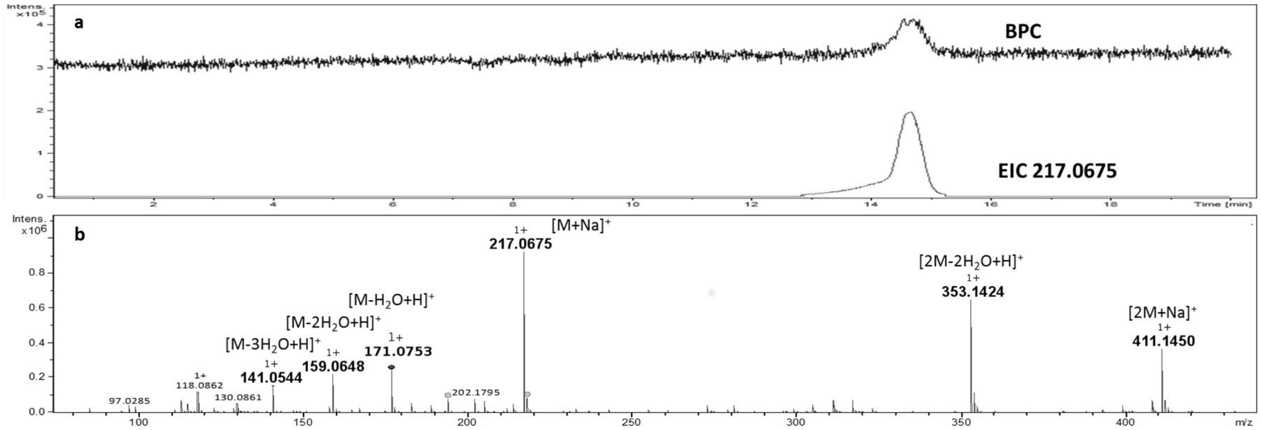

Supplementary Figure 2: HPLC-HRMS chromatogram and MS spectrum of 7dSh.

(**a**) High performance liquid chromatography coupled with high-resolution electrospray ionization mass spectrometry (HR-ESI(+)-MS) of isolated 7dSh (**1**) on a HiPlex Ca column. The base peak chromatogram (BPC) in positive mode and extracted ion chromatogram (EIC) of the isolated inhibitor revealed a mass of 217.0675 ($[M+Na]^+$, $C_7H_{14}O_6Na^+$) and chromatographic purity. (**b**) In the corresponding mass spectrum (R_t = 14.8 min), 7dSh exhibited the characteristic signal of *m/z* = 217.0675 $[M+Na]^+$ ($C_7H_{14}NaO_6^+$). Additional labelled signals correspond to 7dSh fragment ions, which resemble the typical H_2O-loss of carbohydrates and underline the sum formula of 7dSh as $C_7H_{14}O_6$ (molecular weight, M_R = 194.1825).

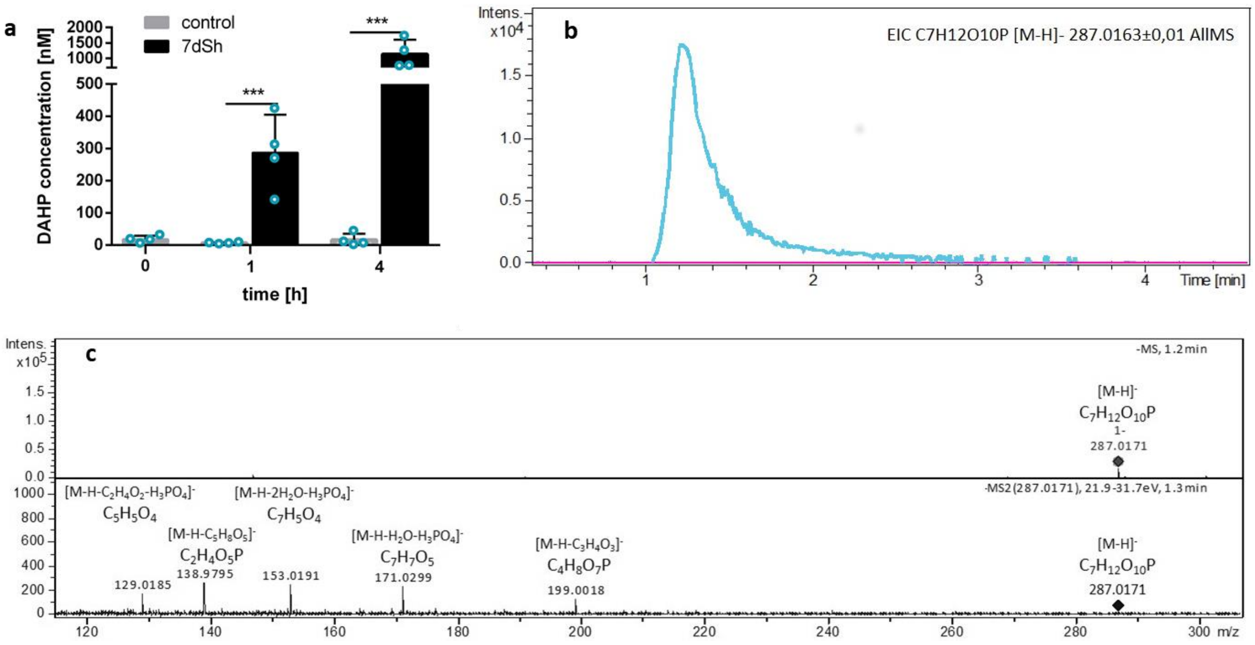

Supplementary Figure 3: DAHP accumulation in 7dSh-treated *A. variabilis* cultures.

Accumulation of 3-deoxy-D-arabinoheptulosonate 7-phosphate (**4**) (DAHP, $C_7H_{13}O_{10}P$) in 7dSh (**1**)-treated (40 µg mL^{-1}, ca. 206 µM) *A. variabilis* cultures (initial OD_{750} = 0.4) determined via LC-HRMS and electrochemical detector (ECD). (**a**) Increase in DAHP in 7dSh-treated *A. variabilis* cultures (detected by ECD). Significant differences between 7dSh treatment and untreated control for each timepoint were analyzed in an unpaired *t*-test (* *p*-value < 0.05; ** *p*-value < 0.01; *** *p*-value < 0.001; NS, not significant). Values represent the mean values of four biological replicates; standard deviations are indicated. Dots indicate data distribution. Source data are provided as a Source Data file. (**b**) LC-HRMS chromatogram with extracted mass signal of DAHP (ESI negative mode: $C_7H_{12}O_{10}P^-$ $[M-H]^-$) of *A. variabilis* culture treated with 7dSh for 1 h (turquoise) and of an untreated control culture (magenta). (**c**) Fragmentation pattern (ESI negative mode) of characteristic DAHP ions.

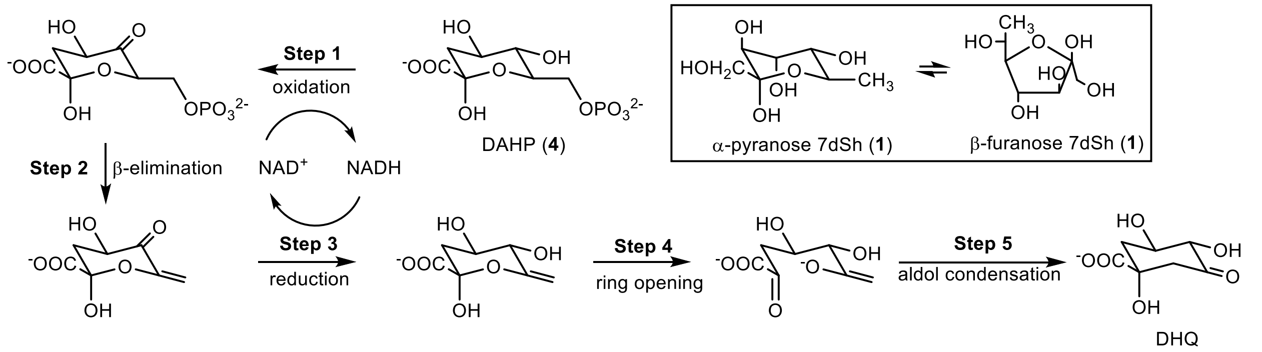

Supplementary Figure 4: Simplified mechanism of the conversion of DAHP to 3-dehydroquinate (DHQ) by DHQ synthase.

Modified from [1]. The pyranose and furanose forms of 7dSh (**1**) are depicted in the box.

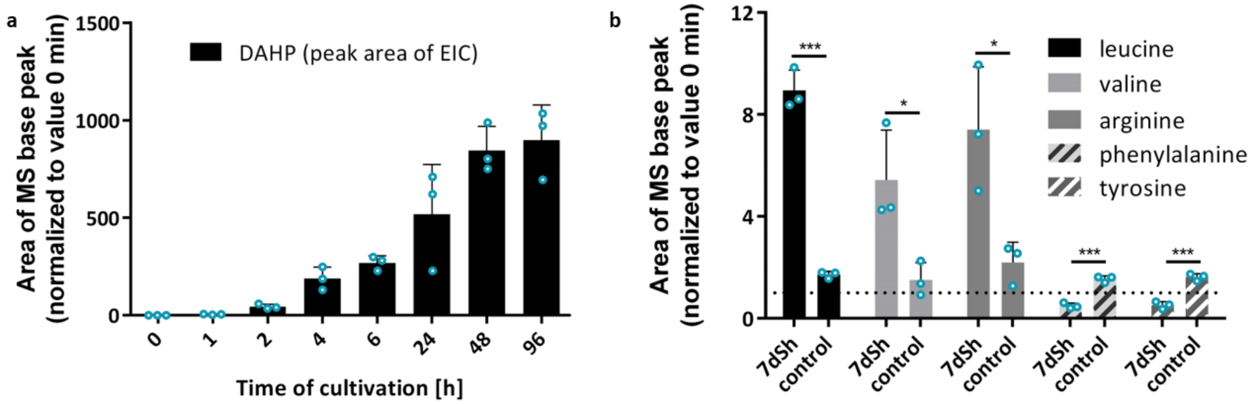

Supplementary Figure 5: Effects of 7dSh on DAHP and amino acid ratios in resuscitating *Synechocystis*. At time 0, nitrogen-starved cultures of *Synechocystis* sp. (initial OD = 0.2) were regenerated by adding $NaNO_3$ (17.3 mM) in the presence of 7dSh (**1**) (40 µg mL^{-1}, ca. 206 µM) or its absence. DAHP (**4**) and amino acid ratios were determined by LC-HRMS and are indicated by the peak areas of extracted ion chromatograms (EIC) normalized to respective value at time 0. (**a**) Increase of DAHP (ESI positive mode: $C_7H_{14}O_{10}P^+$) in 7dSh-treated cultures. (**b**) Changes in amino acid ratios (24 h after addition of $NaNO_3$ and 7dSh). The dashed line indicates the content of respective free amino acids present at time 0 before the addition of $NaNO_3$ and 7dSh. Significant differences between 7dSh treatment and untreated control for each timepoint were analyzed in an unpaired *t*-test (* *p*-value < 0.05; ** *p*-value < 0.01; *** *p*-value < 0.001; NS, not significant). Values in both graphs represent the mean values of three biological replicates; standard deviations are indicated. Dots indicate data distribution. Source data are provided as a Source Data file.

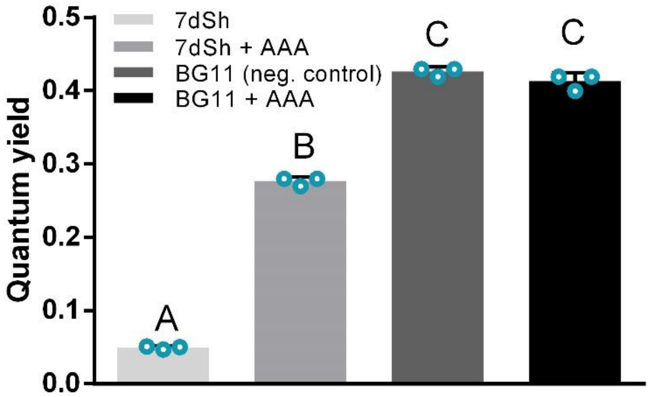

Supplementary Figure 6: Supplementation with aromatic amino acids alleviates effects of 7dSh on *A. variabilis.*

Effect of 7dSh (**1**) (ca. 50 µM) on the quantum yield of *A. variabilis* cultures (initial OD = 0.2) grown for 24 h with or without supplementation with a mixture of aromatic amino acids (AAA; tryptophan, tyrosine, and phenylalanine, 1 mM each; *p*-aminobenzoate and β-hydroxybenzoate, 1 µg mL^{-1} each). Statistical analysis was performed by using a one-way ANOVA. Tukey's multiple comparison test was used as the post-hoc test. Means that were significantly different (*p*-value <0.05) are marked with different capital letters in the diagram. Values represent the mean values of three biological replicates; standard deviations are indicated. Dots indicate data distribution. Source data are provided as a Source Data file.

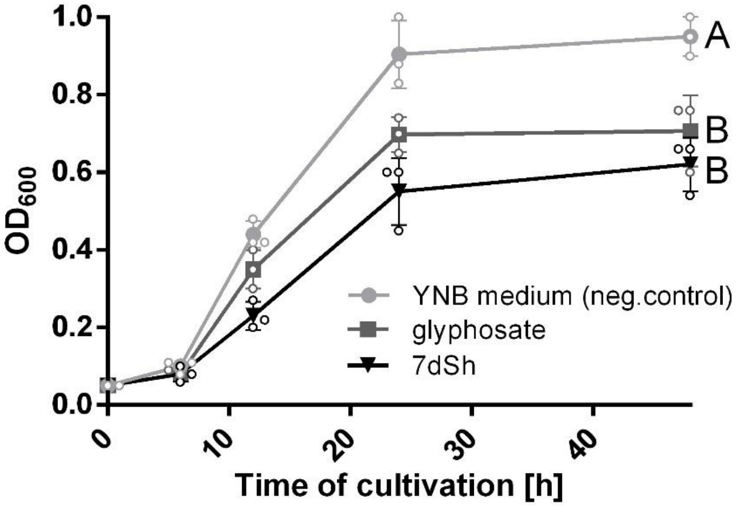

Supplementary Figure 7: Comparison of effects of 7dSh and glyphosate on *S. cerevisiae* grown in minimal medium.

S. cerevisiae was grown in YNB minimal medium in the presence of glyphosate (100 µg mL^{-1}, ca. 590 µM) or 7dSh (**1**) (10 µg mL^{-1}, ca. 50 µM) for 48 h (initial OD_{600} = 0.05). Statistical analysis was performed by using a one-way ANOVA. Tukey's multiple comparison test was used as a post-hoc test to figure out the differences between OD_{600} at timepoint 48h. Means that were significantly different ($p<0.05$) are marked with different capital letters in the diagram. Optical density values represent the mean values of three biological replicates; standard deviations are indicated. Dots indicate data distribution. Source data are provided as a Source Data file.

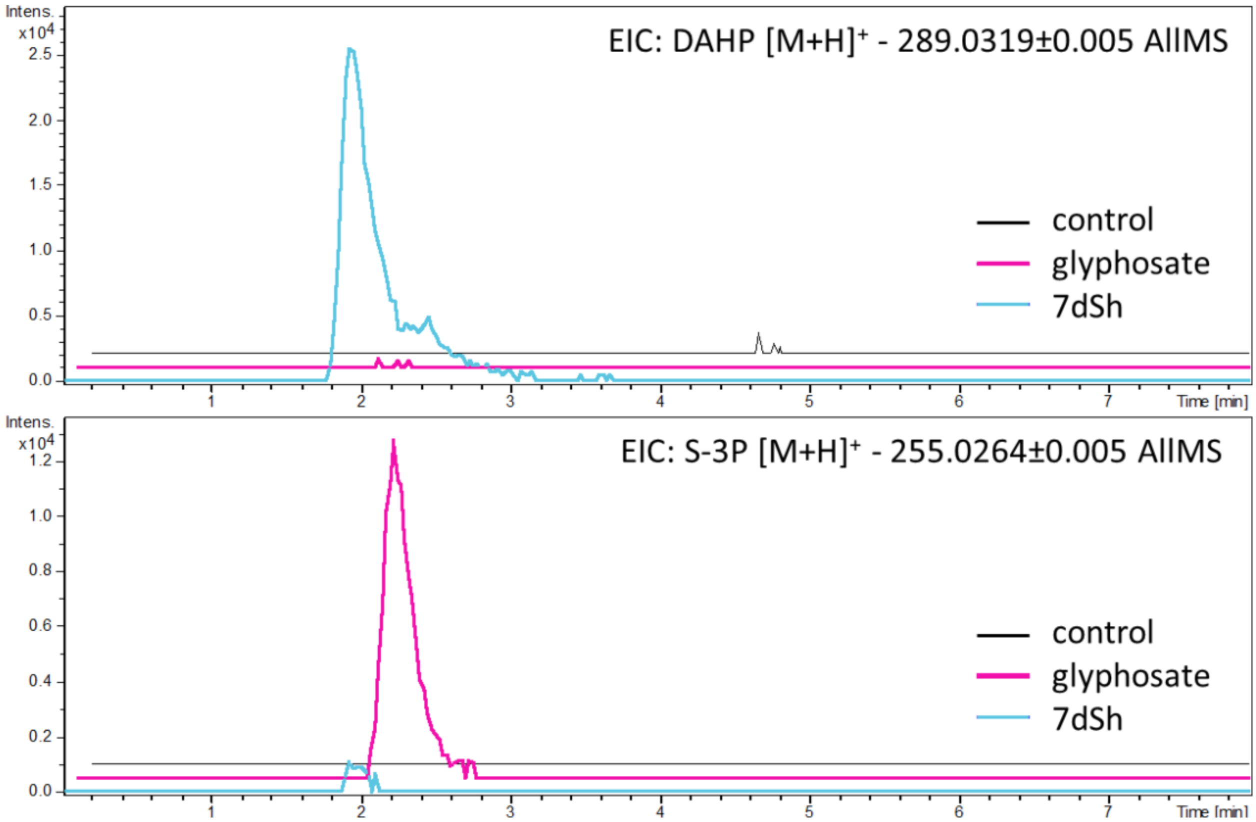

Supplementary Figure 8: LC-HRMS chromatograms show accumulation of shikimate pathway intermediates in *A. thaliana* seedlings treated with 7dSh or glyphosate.

A. thaliana seedlings were grown in presence or absence of 7dSh (**1**) or glyphosate (each 260 µM) for 7 days. The whole plant extracts of 10 seedlings were analyzed by LC-HRMS for the shunt products DAHP (**4**) and shikimate-3-phosphate (S-3P); shown are the stacked EIC chromatograms in positive mode (control in black, shikimate in magenta, 7dSh in turquoise).

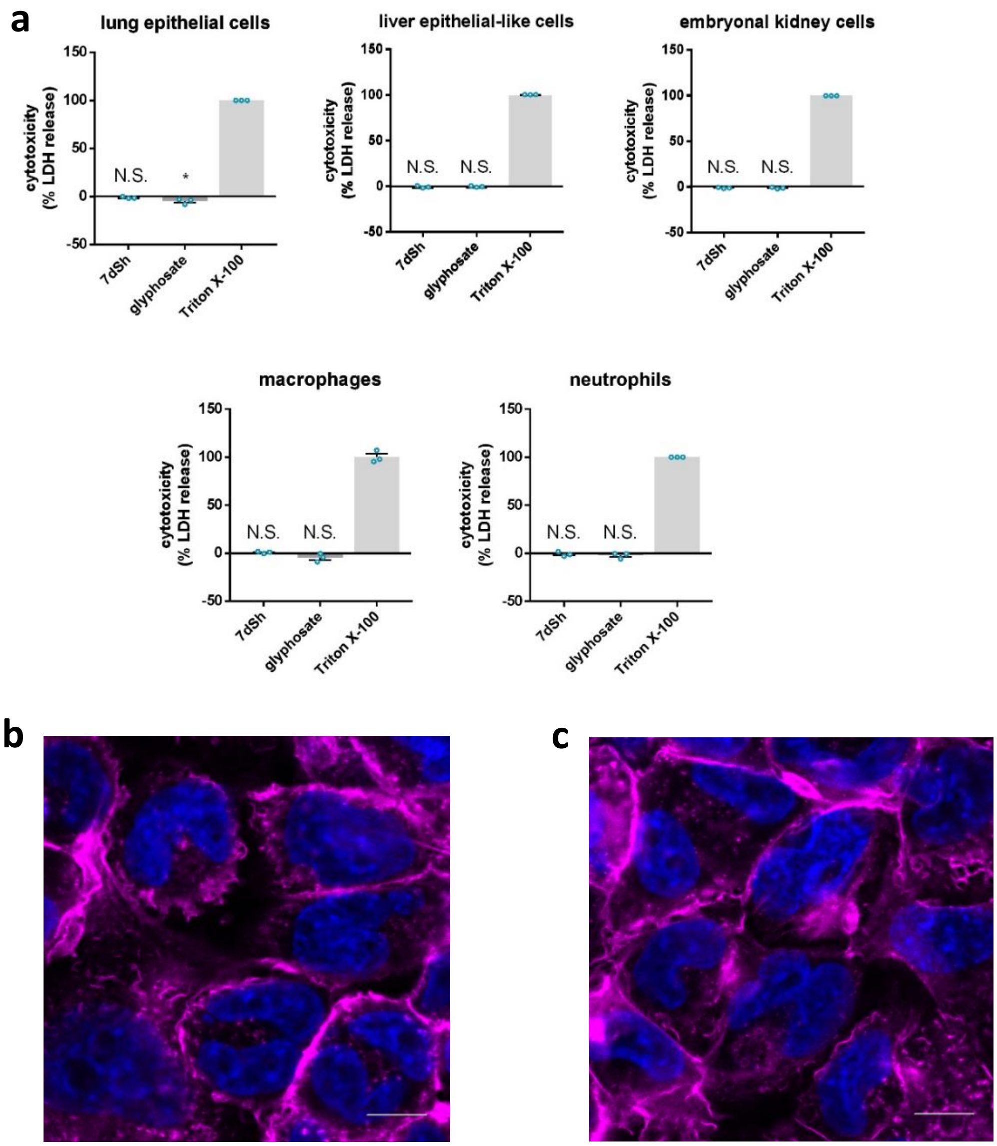

Supplementary Figure 9: **Cytotoxic potential of 7dSh and glyphosate on human cell lines and primary cells.**

(**a**) Effect of 7dSh (**1**) (970 µg mL^{-1}, 5 mM) and glyphosate (845 µg mL^{-1}, 5 mM) on human cell lines and primary cells. Cells were incubated in respective medium with 7dSh or glyphosate. After 24 h (5 h for neutrophils), cytotoxicity was measured according to the release of lactate dehydrogenase and compared to untreated control. The data represent the mean of three independent experiments; standard deviations are indicated. Dots indicate data distribution. Significant differences between 7dSh/glyphosate treatment and untreated control cells were analysed in a one-way ANOVA and a following Tukey's multiple comparisons test (*p-value < 0.05; ** p-value < 0.01; *** p-value < 0.001; N.S., not significant). Source data are provided as a Source Data file. (**b,c**) Cell morphology of untreated human THP1 macrophages (**b**) and those treated with 5 mM 7dSh (**c**) for 24 h. Cells were stained with phalloidin (pink: actin filaments) and DAPI (blue: DNA). Scales bars, 10 µm.

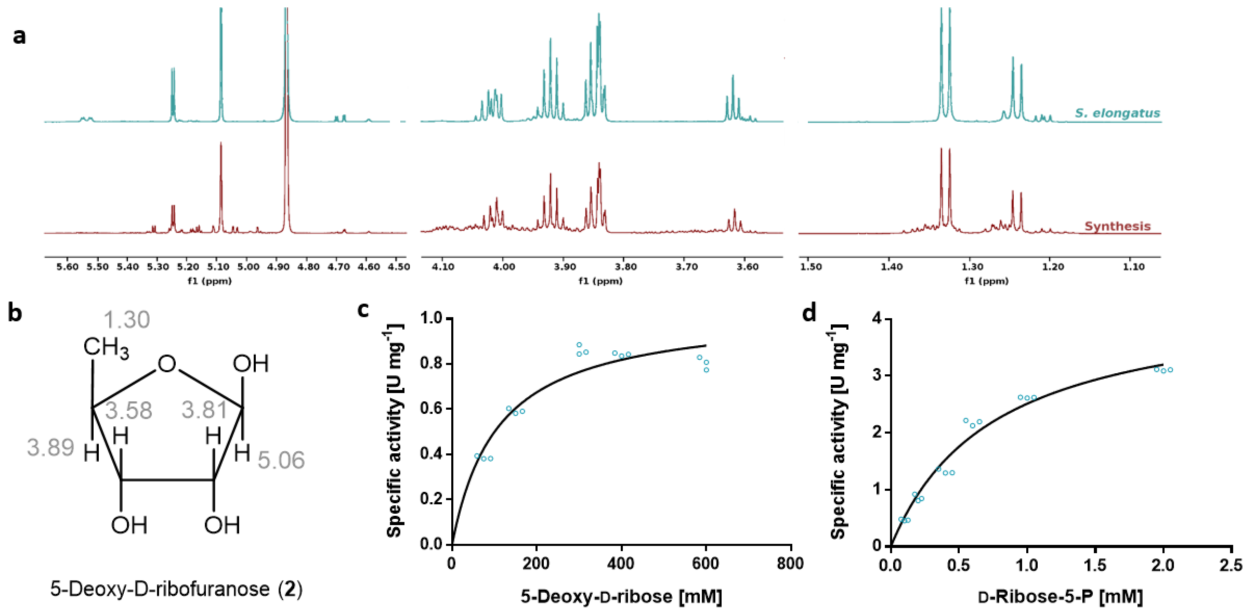

Supplementary Figure 10: ^{1}H NMR analysis of 5-deoxy-D-ribose and enzyme kinetics of the *S. elongatus* transketolase.

a) ^{1}H NMR spectra of 5-deoxy-D-ribose (**2**) (CD_3OD, 600 MHz) purified by chromatography from *S. elongatus* (turquoise) and from chemical synthesis (Glentham Life Sciences) as a control (red). (**b**) Chemical structure of 5-deoxy-D-ribofuranose (**2**) with given assignments (chemical shifts in ppm, gray). (**c**) Michaelis–Menten kinetic profile of the conversion of 5-deoxy-D-ribose by the *S. elongatus* transketolase (K_m = 108.3 mM ± 20.6; V_{max} = 1.04 U mg^{-1} ± 0.06). (**d**) Michaelis–Menten kinetic profile of the conversion of D-ribose 5-phosphate by the *S. elongatus* transketolase (K_m = 0.75 mM ± 0.09; V_{max} = 4.41 U mg^{-1} ± 0.23). Dots in (**c**) and (**d**) represent data distribution of three replicates. Source data are provided as a Source Data file.

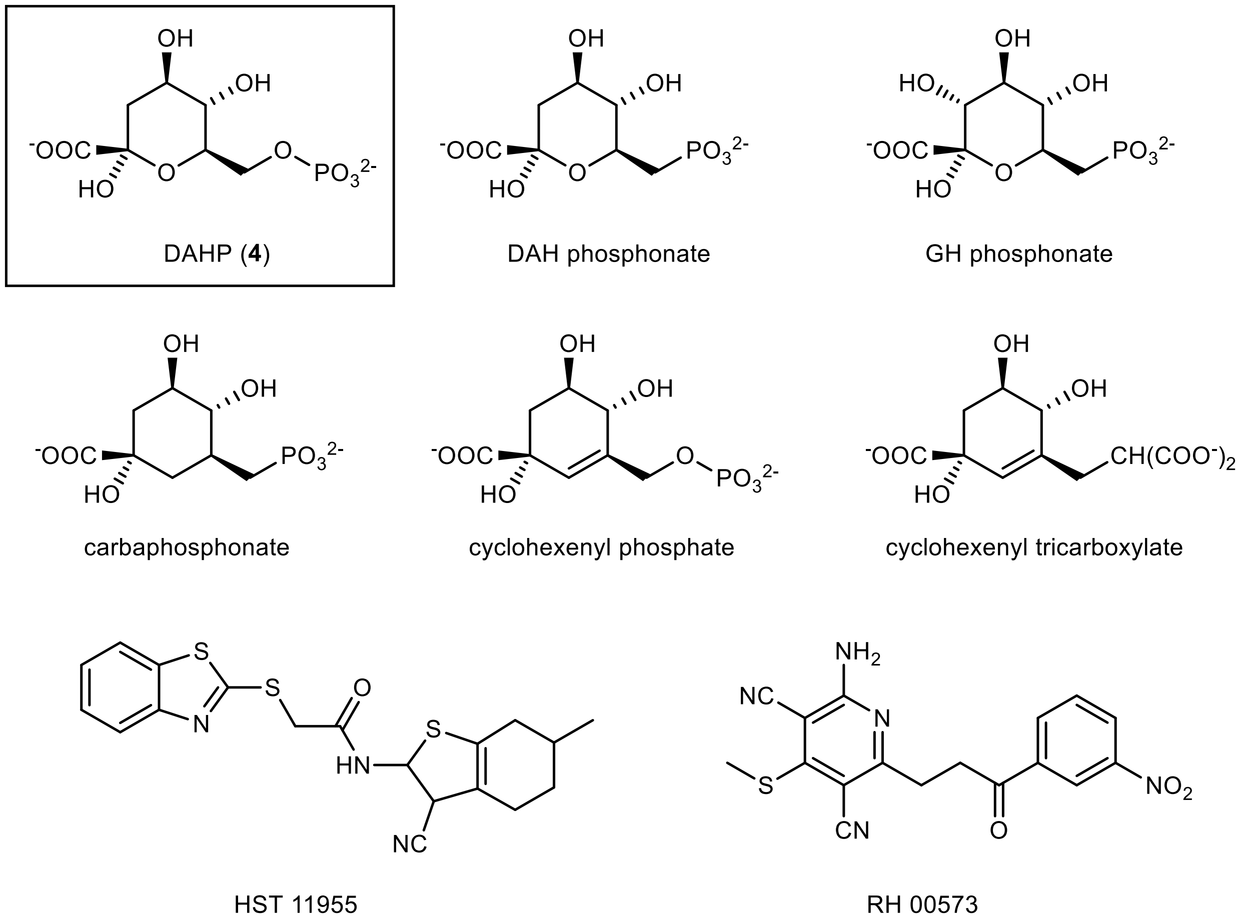

Supplementary Figure 11: Chemical structure of selected known DHQ synthase inhibitors.

The natural 3-dehydroquinate (DHQ) synthase substrate DAHP (**4**) is depicted in the box. DAH phosphonate (3-deoxy-D-*arabino*-heptulosonate 7-phosphonate) and GH phosphonate (D-*gluco*-heptulosonate 7-phosphonate) are phosphonate analogues of DAHP. Carbaphosphonate and its cyclohexenyl derivatives are all substrate analogues of DAHP.

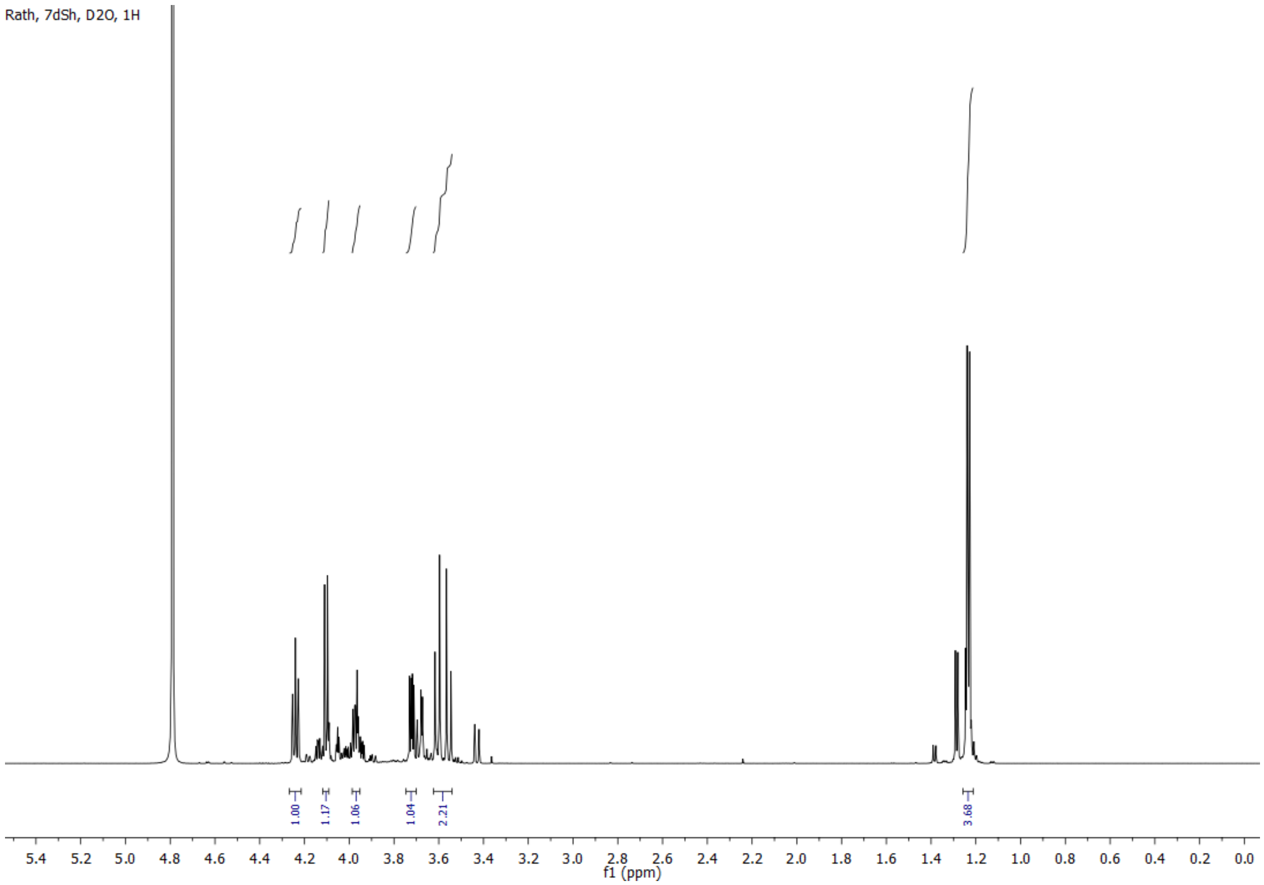

Supplementary Figure 12: ^{1}H NMR spectrum of 7-deoxy-sedoheptulose (7dSh, 1).

D_2O, 298 K, 600 MHz.

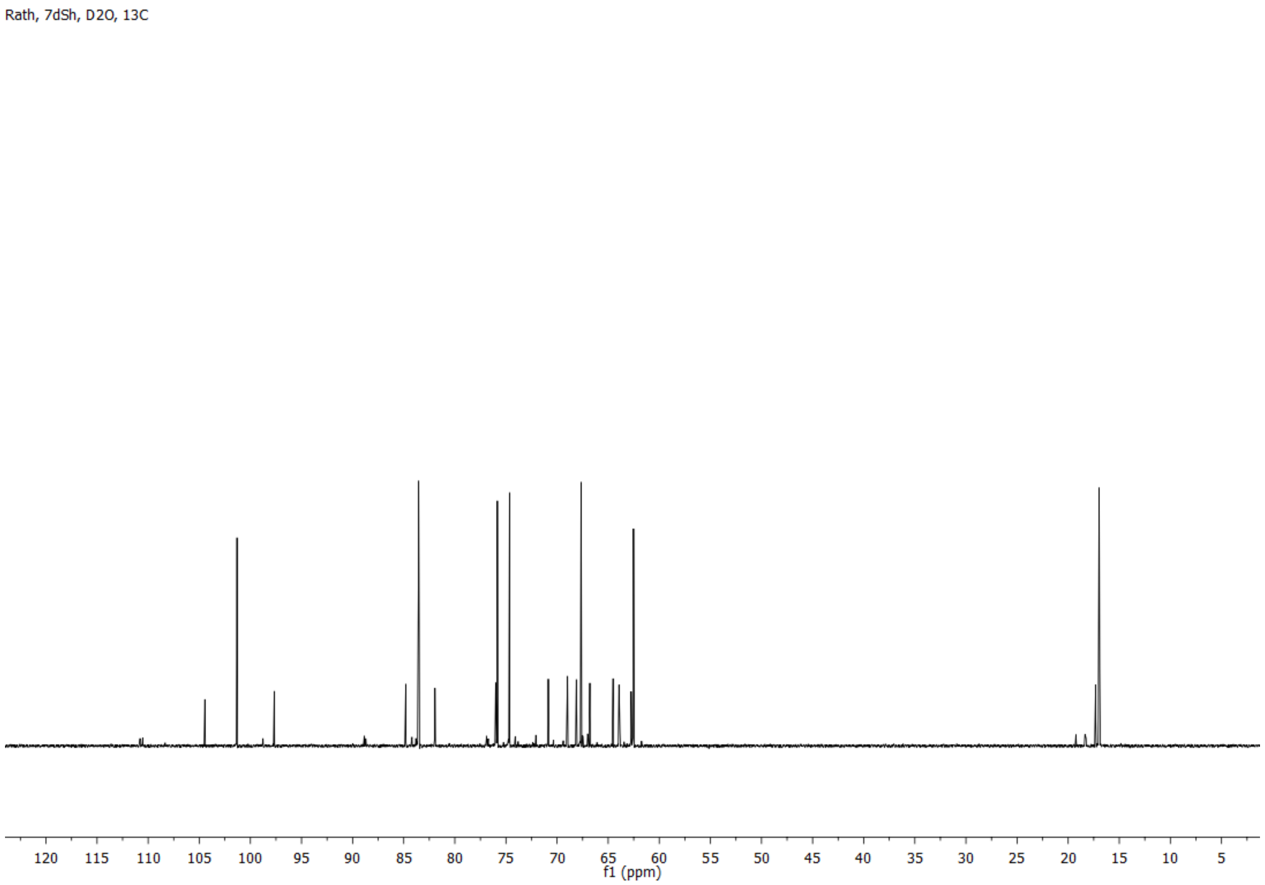

Supplementary Figure 13: ^{13}C NMR spectrum of 7-deoxy-sedoheptulose (7dSh, 1).
D_2O, 298 K, 150.9 MHz.

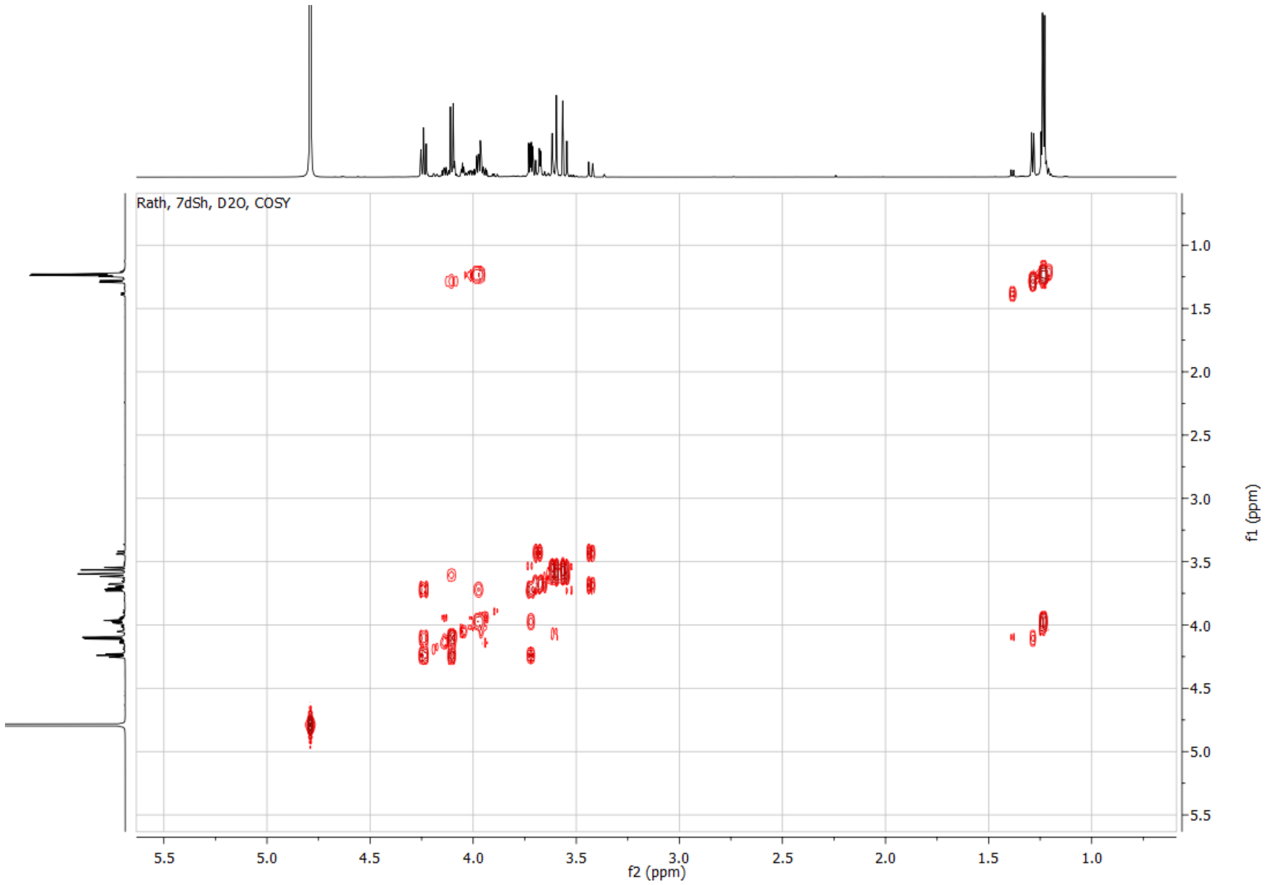

Supplementary Figure 14: H-H-correlation (COSY) spectrum of 7-deoxy-sedoheptulose (7dSh, 1).
D_2O, 298 K, 600 MHz.

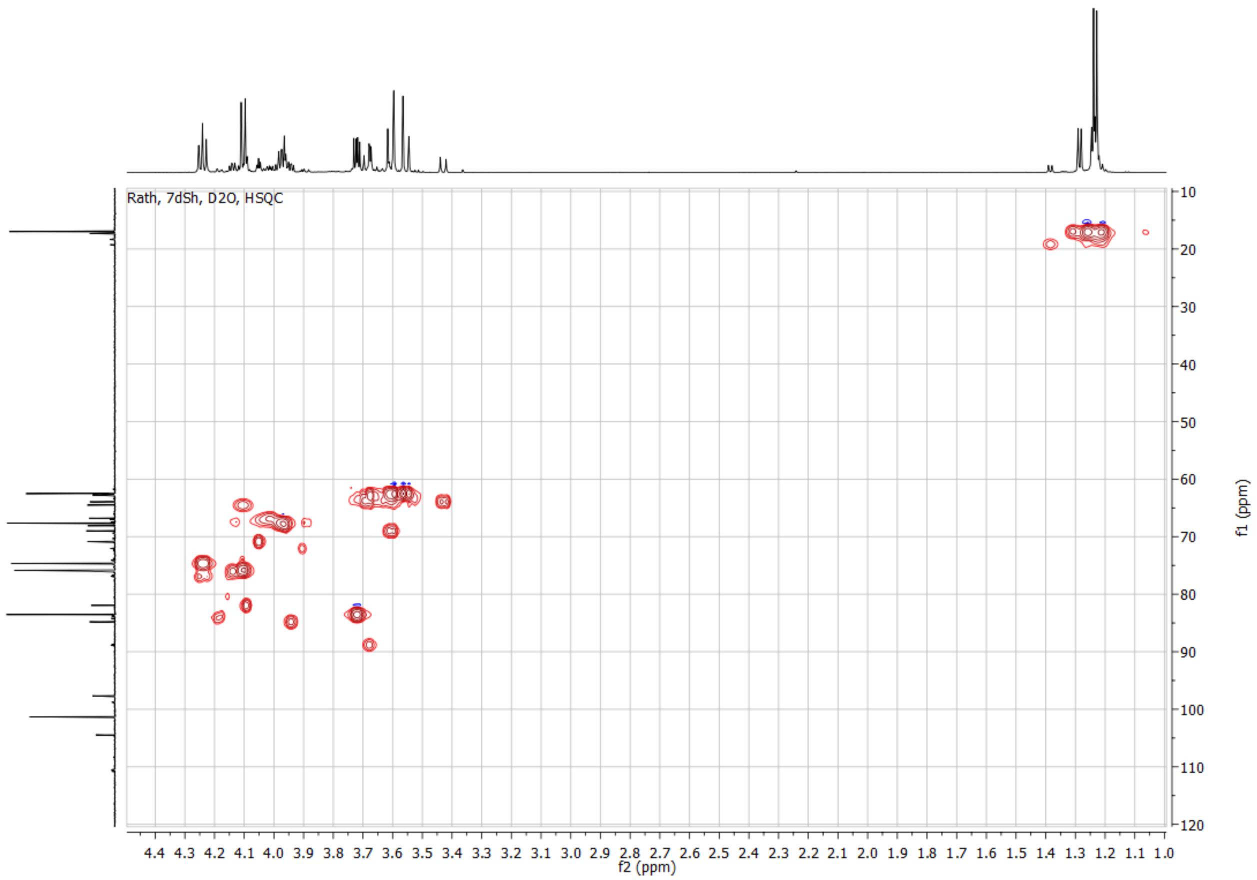

Supplementary Figure 15: CH-correlation (HSQC) spectrum of 7-deoxy-sedoheptulose (7dSh, 1). D_2O, 298 K, 600 MHz (150.9 MHz).

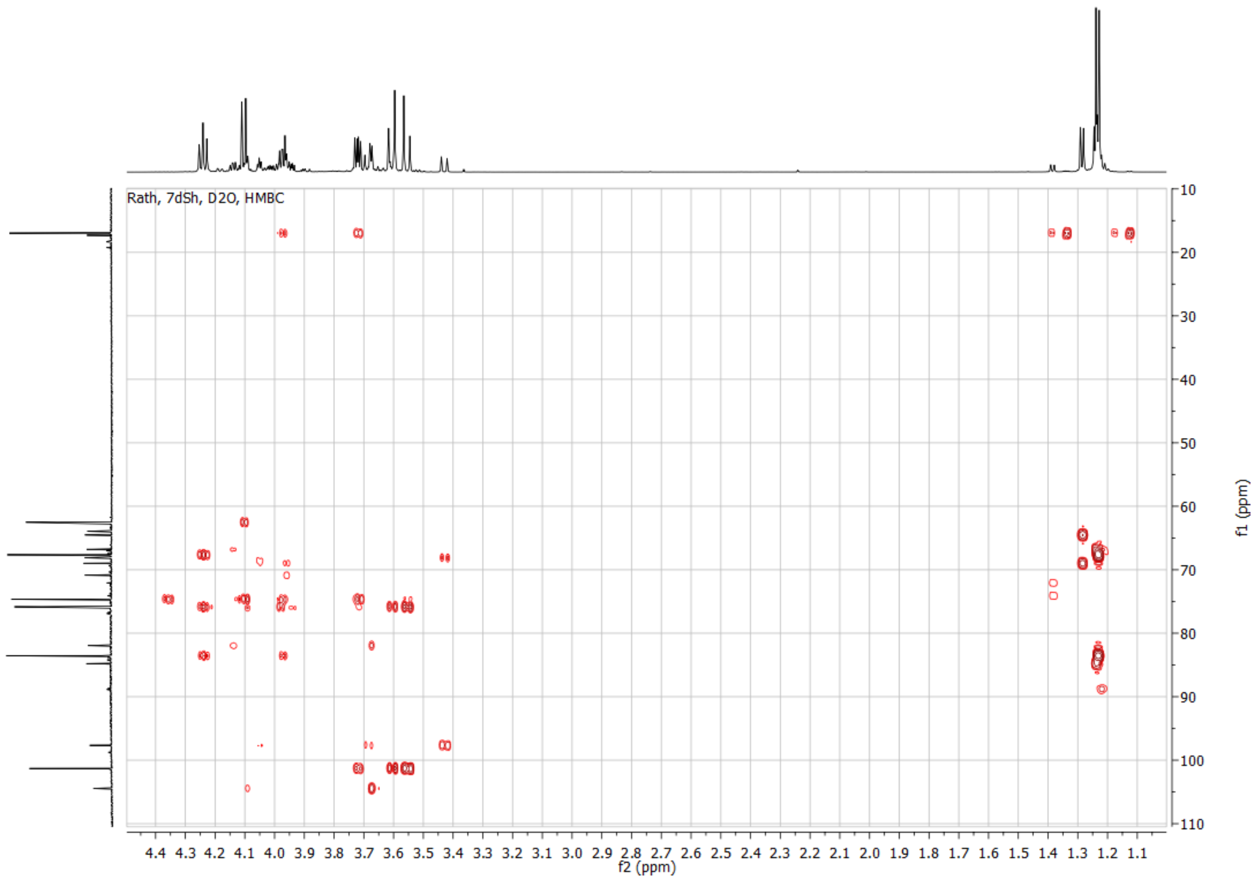

Supplementary Figure 16: Multiple bond CH-correlation (HMBC) spectrum of 7-deoxy-sedoheptulose (7dSh, 1).

D_2O, 298 K, 600 MHz (150.9 MHz).

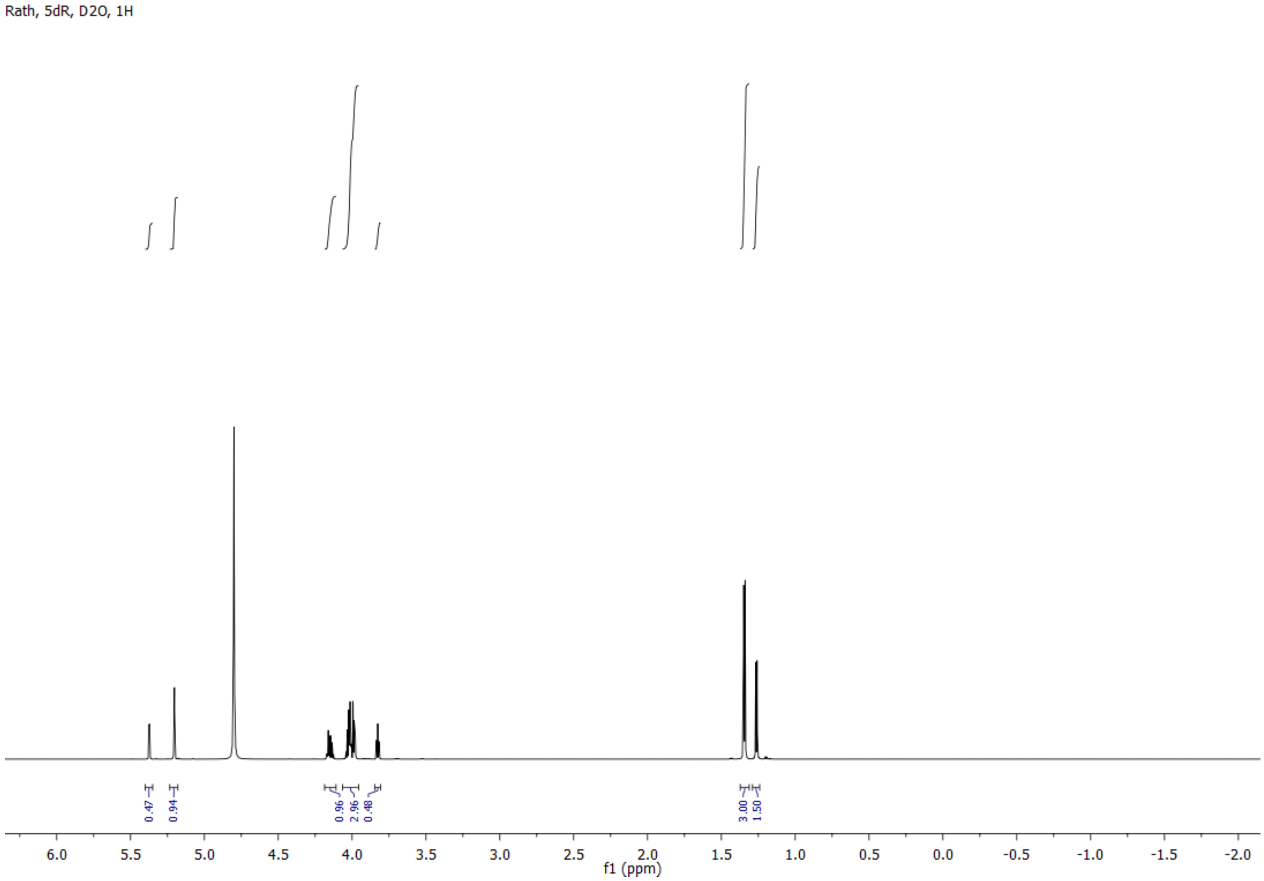

Supplementary Figure 17: ^{1}H NMR spectrum of 5-deoxy-D-ribose (2).
D_2O, 298 K, 700 MHz.

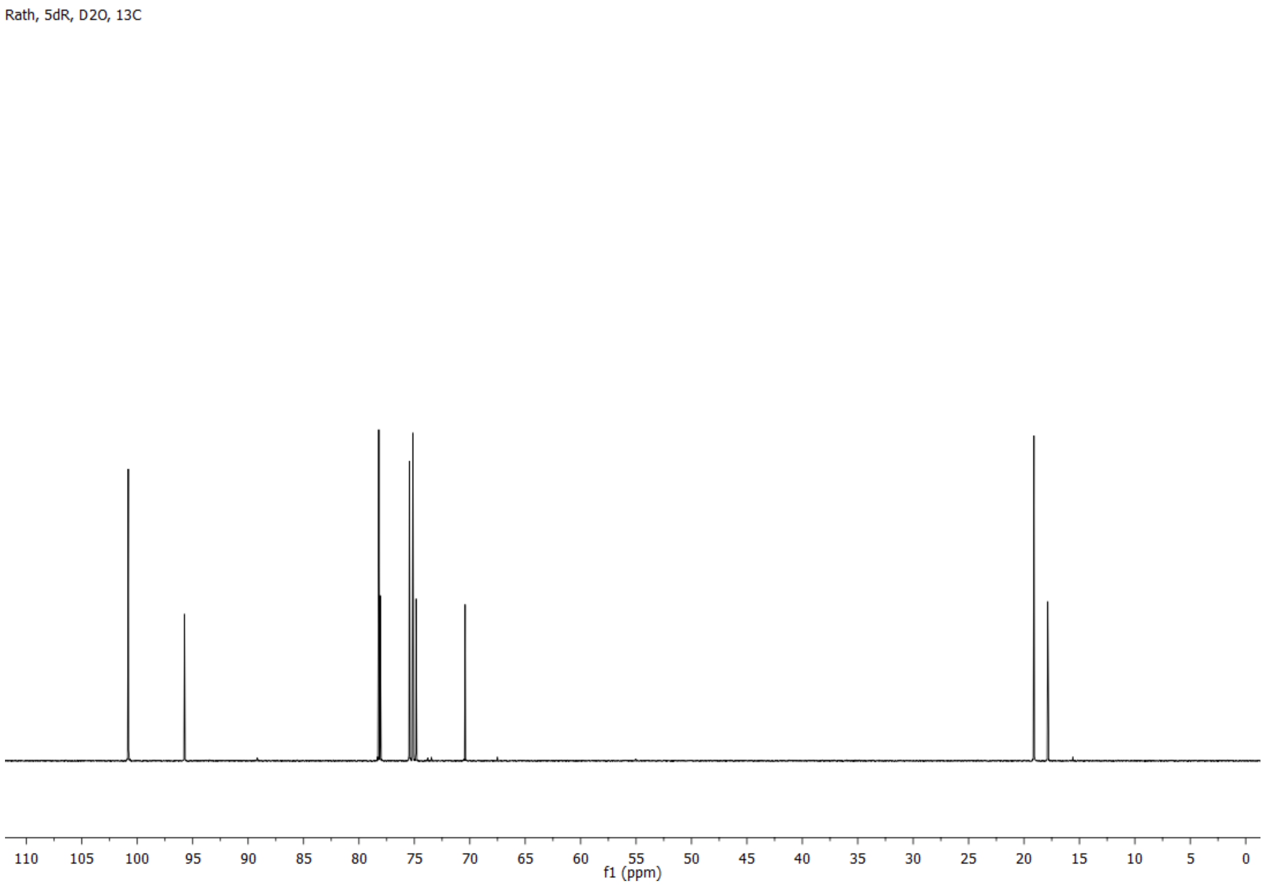

Supplementary Figure 18: ^{13}C NMR spectrum of 5-deoxy-D-ribose (2).
D_2O, 298 K, 176.1 MHz.

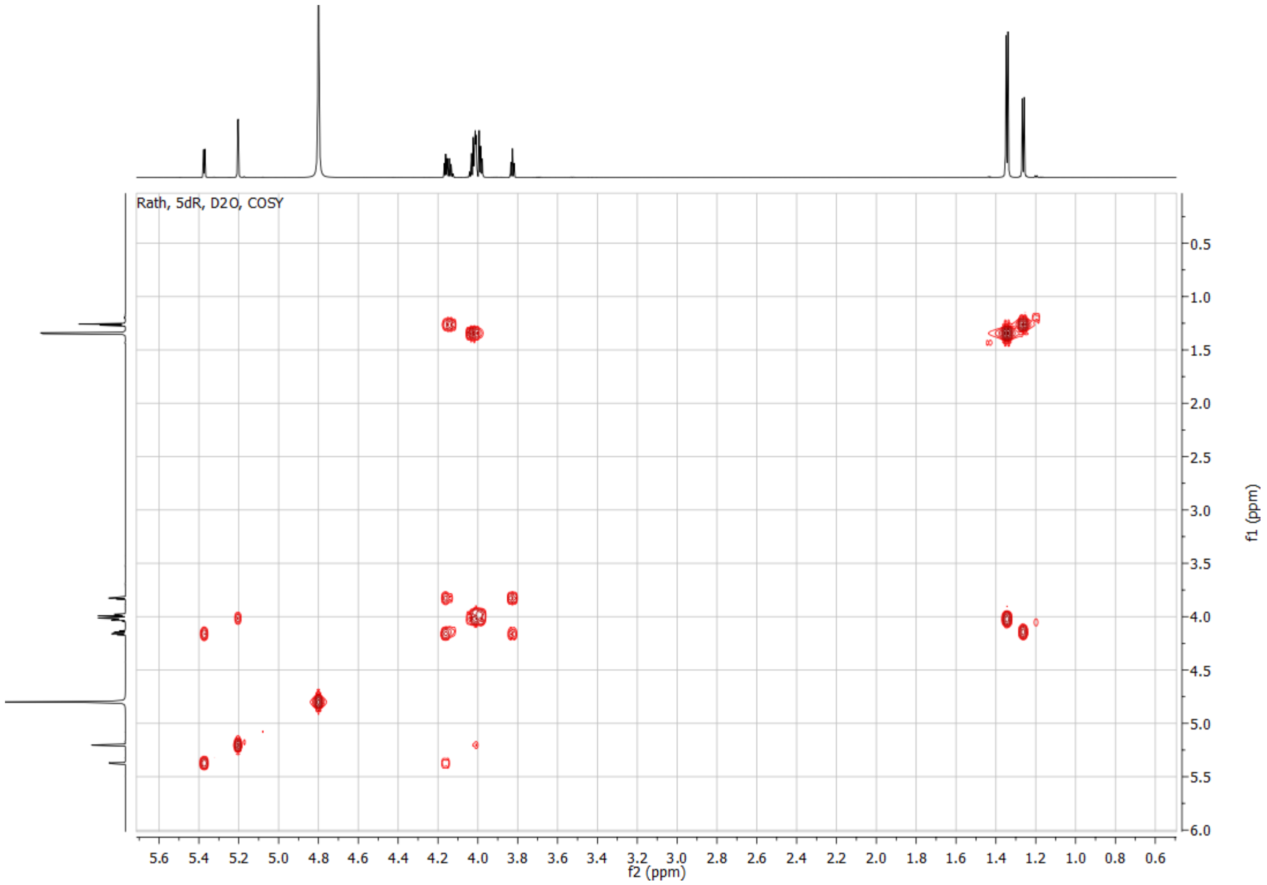

Supplementary Figure 19: H-H-correlation (COSY) spectrum of 5-deoxy-D-ribose (2).
D_2O, 298 K, 700 MHz.

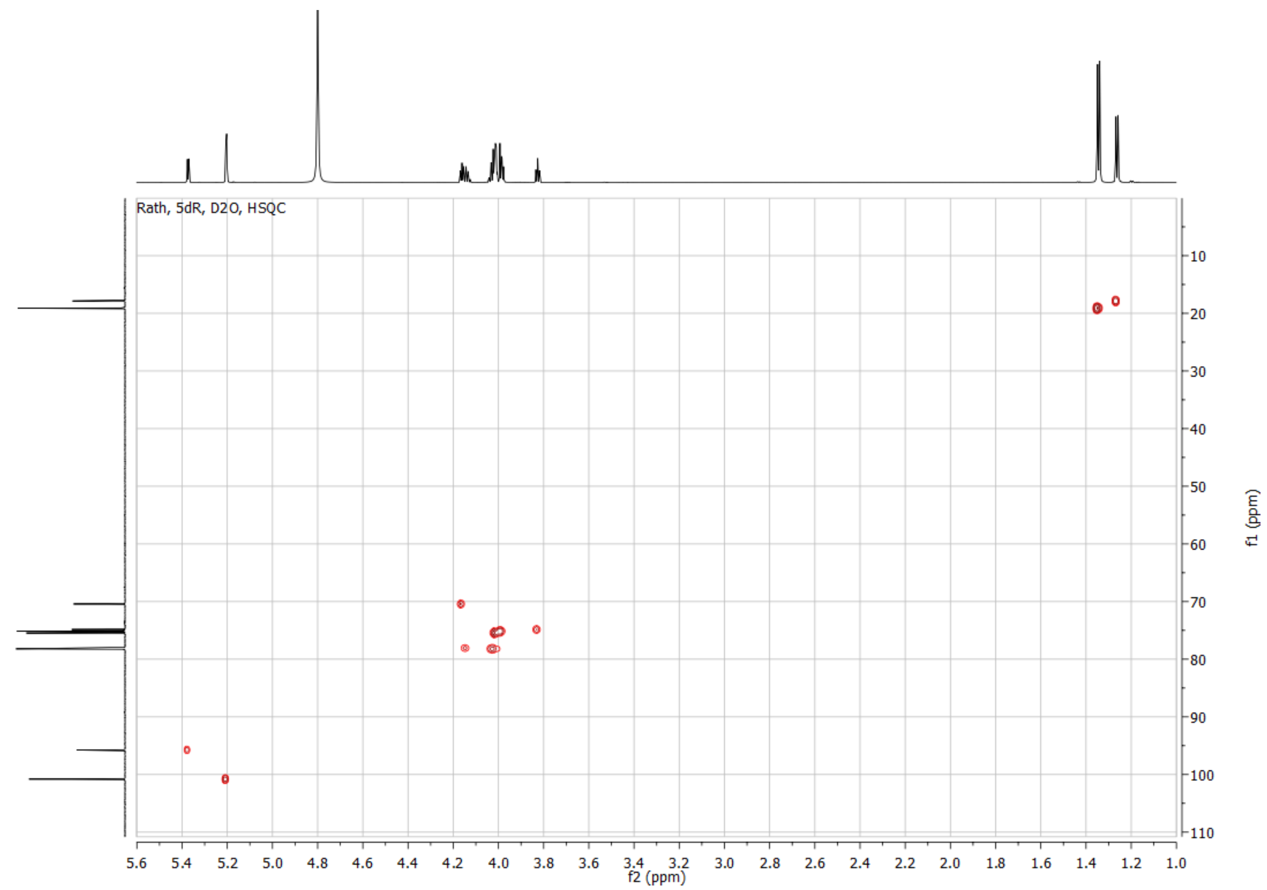

Supplementary Figure 20: CH-correlation (HSQC) spectrum of 5-deoxy-D-ribose (2).
D_2O, 298 K, 700 MHz (176.1 MHz).

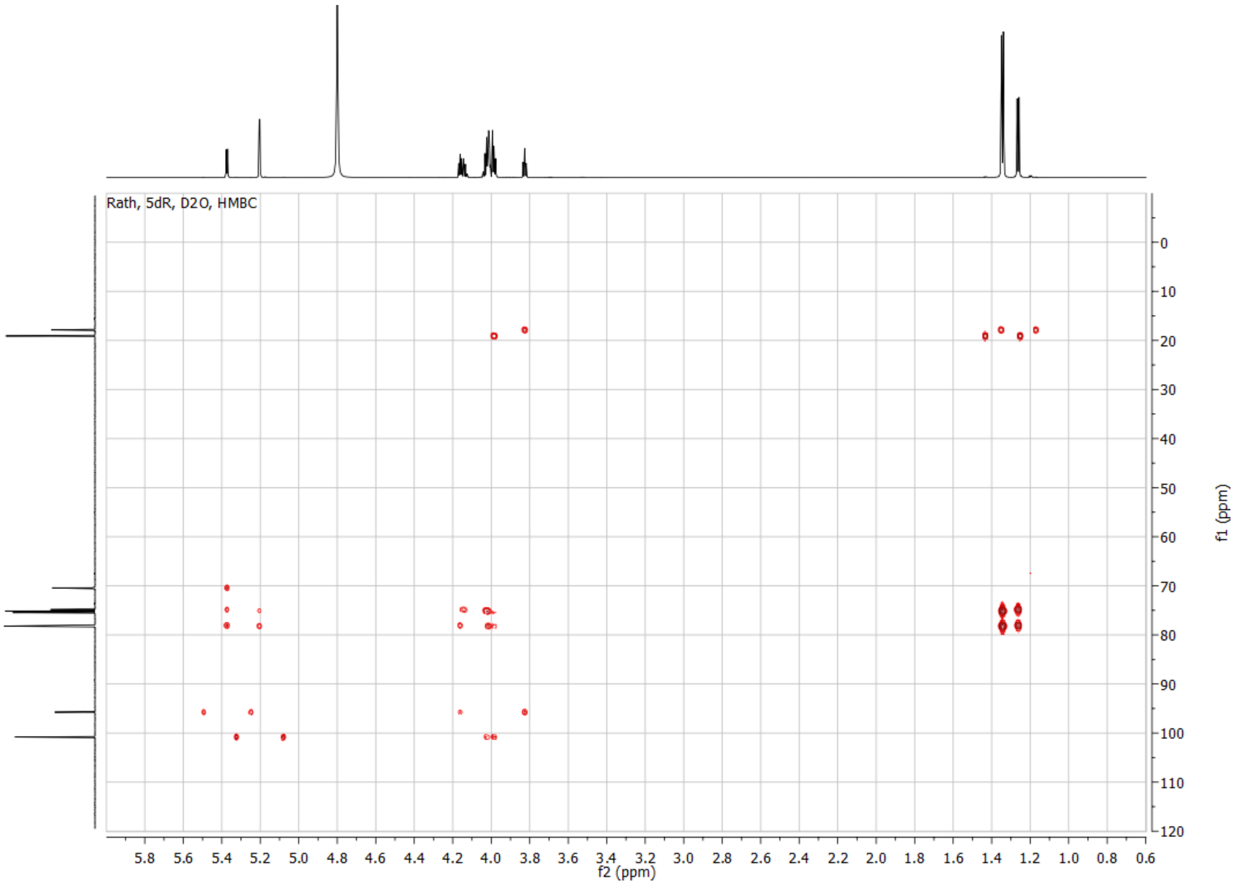

Supplementary Figure 21*:* Multiple bond CH-correlation (HMBC) spectrum of 5-deoxy-D-ribose (2).
D_2O, 298 K, 700 MHz (176.1 MHz).

Supplementary References:

1. Carpenter EP, Hawkins AR, Frost JW, Brown KA. Structure of dehydroquinate synthase reveals an active site capable of multistep catalysis. *Nature* **394**, 299-302 (1998).
